# Ultrasensitive Electrochemical Detection of Butylated Hydroxy Anisole via Metalloporphyrin Covalent Organic Frameworks Possessing Variable Catalytic Active Sites

**DOI:** 10.3390/bios12110975

**Published:** 2022-11-06

**Authors:** Huacong Chu, Xin Sun, Xiaoqian Zha, Shifa Ullah Khan, Yang Wang

**Affiliations:** 1 School of Chemistry and Chemical Engineering, Yangzhou University, Yangzhou 225002, China; 2 The Institute of Chemistry, Faculty of Science, University of Okara, Renala Campus, Punjab 56300, Pakistan

**Keywords:** electrochemical biosensor, covalent organic frameworks, metalloporphyrin, BHA

## Abstract

Three novel two-dimensional metalloporphyrin COFs (MPor−COF−366, M = Fe, Mn, Cu) were fabricated by changing the metal atoms in the center of the porphyrin framework. The physicochemical characteristics of MPor−COF−366 (M = Fe, Mn, Cu) composites were fully analyzed by diverse electron microscopy and spectroscopy. Under optimal conditions, experiments on determining butylated hydroxy anisole (BHA) at FePor−COF−366/GCE were conducted using differential pulse voltammetry (DPV). It is noted that the FePor−COF−366/GCE sensor showed excellent electrocatalytic performance in the electrochemical detection of BHA, compared with MnPor−COF−366/GCE and CuPor−COF−366/GCE. A linear relationship was obtained for 0.04–1000 μM concentration of BHA, with a low detection limit of 0.015 μM. Additionally, the designed sensor was successfully employed to detect BHA in practical samples, expanding the development of COF-based composites in electrochemical applications.

## 1. Introduction

As a synthetic phenolic antioxidant, butylated hydroxy anisole (BHA) is frequently employed in food packaging, biofuels, and pharmaceutical formulations [1]. It has the effect of preventing or delaying the oxidative deterioration of food, thereby improving food stability and prolonging the shelf life of food [2,3]. If the content of BHA exceeds the specified level, it can cause serious health problems in humans, including nutrient loss and even toxic effects [4,5]. Hence, the negative impacts of BHA on human health restrict its application in food goods, and a quick, sensitive, and accurate method to quantify BHA is required.

Several techniques have been developed for determining BHA, including micellar electrokinetic capillary chromatography, gas chromatography, high-performance liquid chromatography, and spectrophotometry [6,7,8,9,10]. However, these methods often encounter difficulties associated with cumbersome or time-consuming procedures. Electrochemical technology has been utilized for the quantitative analysis of BHA owing to its advantages of simple operation, high sensitivity, and rapid response [11,12,13,14]. However, developing advanced materials to achieve high electrochemical performance remains a major research goal.

Covalent organic frameworks (COFs) are formed by powerful covalent links between light elements with a reticular network of molecular building blocks, and exhibit an exceptionally high surface area and high stability, displaying a wide range of applications [15,16,17,18]. Porphyrins are widely used as building blocks for the assembly of covalent organic frameworks because of their high stability, easy chelation with metal ions, and ease of modification [19]. Several porphyrin-based COFs (Por-COFs) have been constructed for use in electro chemiluminescence, heterogeneous catalysis, and photooxidization reactivity [20,21,22]. In addition, metalloporphyrin COFs (MPor-COFs) possess a unique N-C framework, which offers a novel platform for the preparation of self-supporting M-N-C sites with intrinsic metal coordination for use as porous electrocatalysts [23]. However, to our knowledge, the usage of MPor-COFs as electrode materials to improve electrochemical performance has rarely been investigated.

In this work, we synthesized a series of two-dimensional nanomaterials based on MPor−COF−366. Firstly, Por−COF−366 was produced by the condensation reaction of 5,10,5,20-tetra(4-aminophenyl)porphyrin (TAPP) and 1,3,5-benztriformaldehyde (BDA) under acidic conditions. Strong covalent bonds were constructed by using porphyrin macrocycle molecules as building blocks. Then, Por−COF−366 was impregnated with FeSO_4_·6H_2_O, MnSO_4_·4H_2_O and CuCl_2_·6H_2_O, respectively, to obtain the final composites MPor−COF−366 (M = Fe, Mn, Cu). Subsequently, to fabricate an electrochemical sensor, the obtained nanocomposites were fixed on the glass carbon electrode (GCE) surface. The sensor exhibited remarkable catalytic activity and selectivity towards BHA, as the synergistic effect of Por-COFs and metal atoms endowed the sensor with unique properties.

## 2. Materials and Methods

### 2.1. Materials

Chemicals were used exactly as bought and all were of analytical grade. The following substances were obtained from Shanghai Tansoole: 5,10,5,20-tetra(4-aminophenyl)porphyrin (TAPP), 1,3,5-benztriformaldehyde (BDA), 1,4-dioxane, ethanol, tetrahydrofuran, and acetone. Other chemicals were bought from Sinopharm Chemical Reagent Co., Ltd. (Shanghai, China), including CuCl_2_·6H_2_O, FeSO_4_·6H_2_O, MnSO_4_·4H_2_O, CH_2_Cl_2_, methanol, acetic acid, and 1,3,5-trimethylbenzene. As a supporting electrolyte, phosphate buffer solution was prepared using 0.1 M NaH_2_PO_4_/Na_2_HPO_4_. 

### 2.2. Synthesis of Por−COF−366, MPor−COF−366 (M = Fe, Mn, Cu)

Por−COF−366 was synthesized according to a previously reported method with some modifications [24]. TAPP (18 mg, 0.025 mM), BDA (10 mg, 0.075 mM), 1.0 mL ethanol, 1.0 mL mesitylene, and 0.25 mL acetic acid (6 M) were added into a 5 mL glass bottle. The mixed solution was ultrasonically dispersed for 5 min, followed by stirring at room temperature for 15 min, then transferred to a Teflon-sealed reactor and heated to 120 ℃ (72 h). Following the reaction, the product was filtered and collected, then washed with 1, 4-dioxane, THF, and acetone, respectively, and finally dried under vacuum at 40 ℃ for 12 h to obtain Por−COF−366.

Por−COF−366 (20.0 mg) and FeSO_4_·7H_2_O (25 mg, 0.1 mM) were dissolved in a mixture of 12 mL dichloromethane and methanol. The mixture was stirred continuously for 24 h. After centrifugation, the composites were washed with CH_2_Cl_2_ and were dried under vacuum at 60 °C for 12 h to obtain the product FePor−COF−366. After replacing the metal salts with MnCl_2_·6H_2_O and CuCl_2_·6H_2_O, MnPor−COF−366 and CuPor−COF−366 were obtained by the same synthesis process.

### 2.3. Preparation of MPor−COF−366 (M = Fe, Mn, Cu) Modified Electrode

GCE was meticulously polished with α-Al_2_O_3_ before being sequentially rinsed in water and ethanol and then dried under N_2_. The GCE surface was coated with 5.0 μL of FePor−COF−366 (1 mg/mL) suspension, and dried using infrared light. After that, 2.0 μL 5 % chitosan solution was selected as binder. MnPor−COF−366/GCE, CuPor−COF−366/GCE, and Por−COF−366/GCE were all modified using the same method.

### 2.4. Preparation of Samples

Rapeseed, corn, and peanut oils were bought at a nearby grocery. Then, 1.0 g of the material was dissolved in 10 mL of ethanol. The product was centrifuged after being oscillated for 10 min. The supernatant was collected, and filtered through a Millipore nylon filter with a 0.22 μm pore size. A 1.0 mL aliquot of the final product was put into 9.0 mL of pH 4.0 PBS for further analysis.

### 2.5. Characterizations

The morphology of the composites was examined using transmission electron microscopy (TEM, Tecnai 12, 120 KV, Amsterdam, Netherlands) and scanning electron microscopy (SEM, S-4800II, Tokyo, Japan). A Cary 610/670 infrared microspectrometer (Varian, Palo Alto, Santa Clara, CA, USA) was employed to obtain the FTIR spectra. Utilizing a D8 Advance X-ray diffractometer (Bruker Co., Karlsruhe, Germany), the X-ray diffraction (XRD) patterns were gathered from 1.5° to 80° at ambient temperature. XPS testing was carried out using an ESCALAB 250Xi XPS spectrometer from Thermo Scientific, Massachusetts, USA with an aluminum Kα X-ray source.

### 2.6. Electrochemical Analysis

A CHI852C electrochemical analyzer (Shanghai Chenhua Co., Shanghai, China) was applied for all electrochemical tests. A standard three-electrode system was employed to perform cyclic voltammetry (CV) and differential pulse voltammetry (DPV).

## 3. Results and Discussions

### 3.1. Characterizations of Synthesized Composites

**Figure 1:**
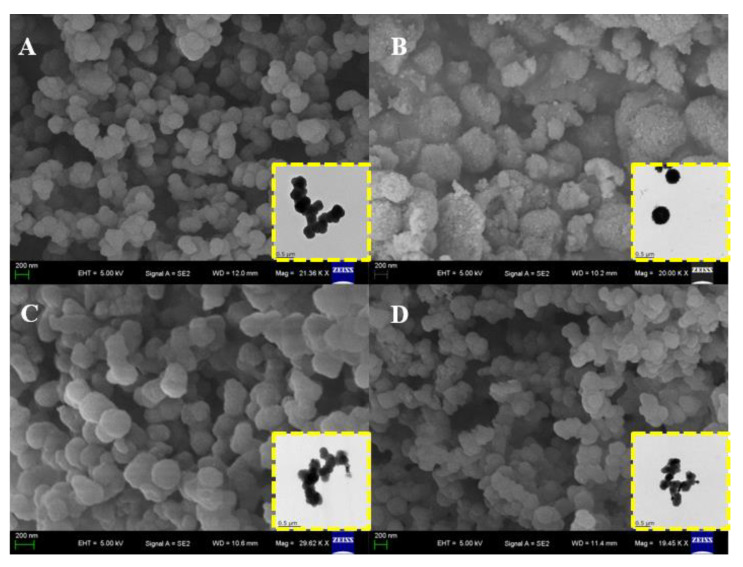
SEM and TEM (inset) images of (**A**) Por−COF−366, (**B**) FePor−COF−366, (**C**) MnPor−COF−366, and (**D**) CuPor−COF−366.

The morphologies of Por−COF−366 and MPor−COF−366 composites were characterized using SEM and TEM measurements. In Figure 1A, it is shown that Por−COF−366 is a generally spherical nanomaterial with a size of 180 nm on average. After coordination with metal ions, the surfaces of MPor−COF−366 composites became rough, but the morphology did not change significantly.

**Figure 2:**
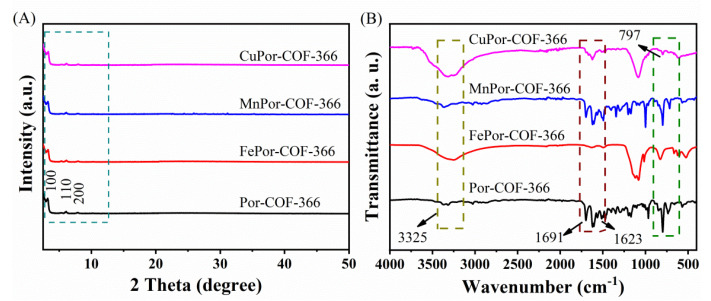
(**A**) XRD patterns and (**B**) FTIR spectra of Por−COF−366 and MPor−COF−366 (M = Fe, Mn, Cu).

To further confirm the structure and crystallinity of the produced Por−COF−366 and MPor−COF−366, spectral PXRD analysis was performed. As displayed in Figure 2A, the obvious characteristic diffraction peak at 2θ = 3.5° was attributed to the (100) plane. Other weaker peaks appeared at 2θ = 6.1° and 7.9°, corresponding to the (110) and (200) planes, respectively, which were consistent with the reported data [25]. In addition, Por−COF−366 and MPor−COF−366 composites exhibited sharp pyrrole ring vibration at 797 cm^−1^, and the characteristic stretching vibrations of C=N at 1623 cm^−1^ were seen in FTIR spectrum (Figure 2B). There were also absorbance peaks at 1691 cm^−1^ and 3325 cm^−1^, which were attributed, respectively, to the residual C=O and stretching N-H vibration of residual -NH_2_ groups [26].

**Figure 3:**
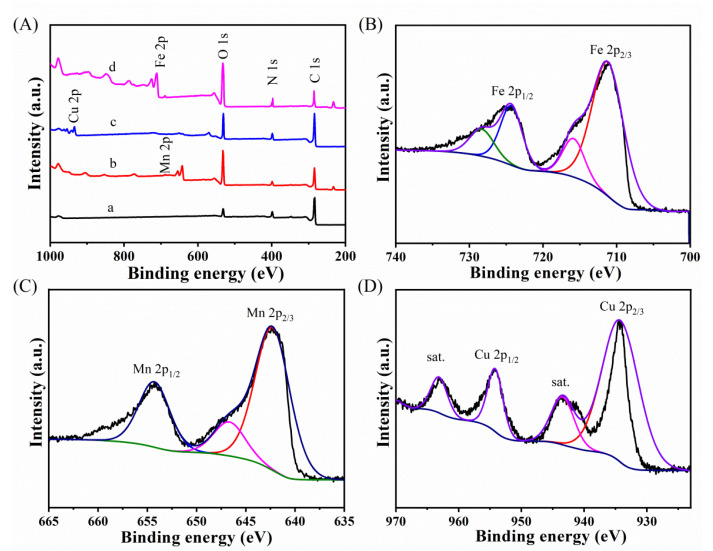
(**A**) Full spectrum diagram of (a) Por−COF−366, (b) MnPor−COF−366, (c) CuPor−COF−366, and (d) FePor−COF−366. XPS analysis of (**B**) Fe 2p, (**C**) Mn 2p, and (**D**) Cu 2p.

Furthermore, Figure 3 shows the XPS measurements to evaluate the chemical state of the as-synthesized Por−COF−366 and MPor−COF−366 composites. The survey spectrum is shown in Figure 3A, revealing that the composites were composed of Fe, Mn, Cu, N, O, and C species. Figure 3B shows the spectrum of Fe 2p. The FePor−COF−366 was successfully synthesized, as evidenced by the four main peaks at 711.4, 715.9, 724.5, and 728.5 eV, which were consistent with the Fe^2+^ 2p_3/2_, Fe^3+^ 2p_3/2_, Fe^2+^ 2p_1/2_, and Fe^3+^ 2p_1/2_, respectively [27,28]. Additionally, Mn 2p in the XPS spectra shown in Figure 3C contained peaks at around 642.3 and 654.6 eV that were attributed to Mn 2p_2/3_ and Mn 2p_1/2_, respectively. Manganese ions were found to exist as Mn^4+^ with spin-orbital splitting of 12.3 eV [29]. Another peak at 646.7 eV was assigned to Mn^3+^, indicating that the process may have created Mn in a lower oxidation state [30]. Additionally, as depicted in Figure 3D, the typical peaks at 954.2 and 934.4 eV corresponded to Cu 2p_1/2_ and Cu 2p_3/2_, respectively [31]. Typical peaks of the Cu(II) state were observed as two satellite peaks at about 963.12 and 943.46 eV [32]. These findings agreed with those of the XRD and FTIR analyses.

### 3.2. Electrochemical Properties

**Figure 4:**
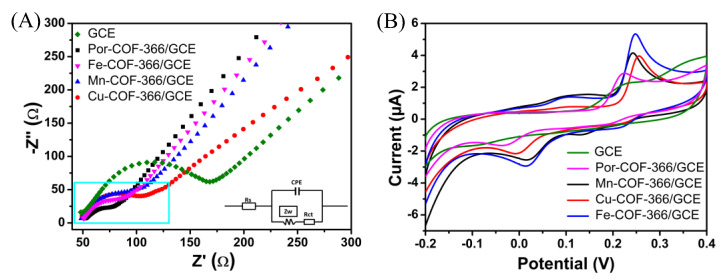
(**A**) EIS of bare GCE, Por−COF−366, and MPor−COF−366 (M = Fe, Mn, Cu). (Inset is the Randle circuit model). (**B**) CV values of bare GCE, Por−COF−366, and MPor−COF−366 (M = Fe, Mn, Cu) in 0.1 M PBS (pH 4.0) with the presence of 50 μM BHA at a scan rate of 100 mV s^−1^.

The changes between the electrode interface and the solution were investigated using electrochemical impedance spectroscopy (EIS) in 5.0 mM [Fe(CN)_6_]^3−/4−^ electrolyte [33]. The characteristic EIS plots of the different electrodes are shown, and an equivalent circuit (Randles model) is provided in the inset of Figure 4A. The Randles equivalent electrical circuit model consists of the electrode surface resistance (Rs), the element of interfacial electron transfer resistance (Rct), Warburg impedance (Zw), and the constant phase angle element (CPE). In the Nyquist impedance plot, a straight line at low frequencies was associated with a diffusion-limited process, and a small semicircle at high frequencies was associated with an electron-transfer-limited process. The semicircle is equal to the value of Rct, and the GCE revealed the largest Rct value (166.5 Ω). After immobilizing the Por−COF−366, the Rct value decreased to 77.2 Ω, indicating that the presence of Por−COF−366 lowered the charge transfer barrier, which may have been caused by the π-π stacking interaction between Por−COF−366. Because electrons were delocalized on all conjugated nanocomposites, the mobility of electrons was effectively enhanced. The Rct values of the MPor−COF−366 modified electrodes followed the orderFePor−COF−366 (88.6 Ω) < MnPor−COF−366 (96.7 Ω) < CuPor−COF−366 (104.1 Ω), indicating that MPor−COF−366 can effectively improve electron transfer and electrochemical activity.

The electrochemical behaviours of GCE, Por−COF−366/GCE, and MPor−COF−366/GCE were investigated through CV. As exhibited in Figure 4B, the redox peaks in the modified electrodes were well-defined and the electrochemical activity was significantly improved compared with the weak redox peaks on GCE. The anodic peaks located at 0.106 and 0.247 V belonged to butylated hydroquinone and BHA, respectively. The cathodic peak at 0.014 was assigned to *tert*-butylquinone (TBQ). Compared with CuPor−COF−366/GCE and MnPor−COF−366/GCE, a narrowed redox peak potential separation (Δ*E_p_*) and enhanced peak current value were observed after modification with FePor−COF−366/GCE. The Δ*E_p_* value in a cyclic voltammogram is one of the important factors for estimating the electron charge-transfer rate at the electrode–electrolyte interface. Generally, the Δ*E_p_* value is inversely proportional to the charge-transfer rate constant. Here, the Δ*E_p_* values decreased in the sequence CuPor−COF−366/GCE (263.5 mV) > MnPor−COF−366/GCE (232.5 mV) > FePor−COF−366/GCE (229.5 mV). The results indicated that the engineered FePor−COF−366 largely minimized the electron-transfer barrier between the redox species and the electrode. The peak currents of the various modified electrodes followed the order FePor−COF−366 > MnPor−COF−366 > CuPor−COF−366 > Por−COF−366 > bare GCE, indicating that the introduction of Por−COF−366 and MPor−COF−366 can effectively improve the electrochemical activity of the electrode. Among all the investigated samples, the FePor−COF−366 exhibited the best electrochemical response. This phenomenon may be due to the fact that atomically dispersed Fe in the porphyrin macrocycle was coordinated as the catalytically active centre, which was fully exposed to the interaction with BHA, providing a greater specific surface area, increasing the quantity of open active sites, and improving the speed of electron transfer in the composite.

### 3.3. Optimization of the Experimental Parameters

**Figure 5:**
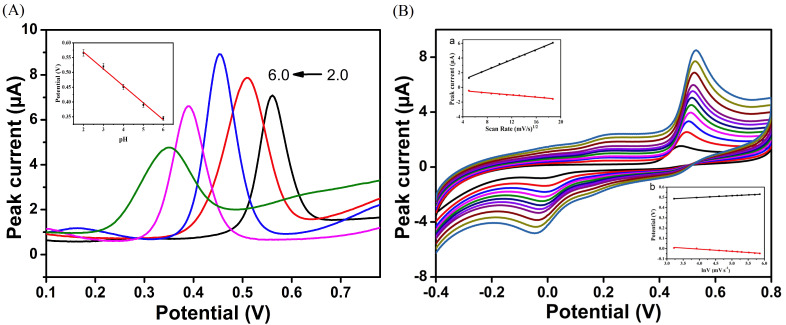
(**A**) Effect of pH on the peak currents of BHA, the inset is the linear relationship between pH values and electric potential; (**B**) CV values of the FePor−COF−366/GCE in 0.1 M PBS (pH 4.0) containing 50 μM BHA at different scan rates, the insets are (**a**) the linear relationship between currents and sweep-speed square root, and (**b**) the linear relationship between redox peak potentials (*E*_p_) and lnν.

Figure 5A depicts the results of investigation into the effect of pH values on the DPV response of BHA, for FePor−COF−366/GCE over a potential range of 0.1 to 0.8 V at various pH ranges from 2.0 to 6.0. With the increase of solution pH, the oxidation peak current of BHA showed first an increasing trend and then a decrease. The maximum current of BHA was reached at pH 4.0. At pH values between 4.0 and 6.0, a decrease was observed in the oxidation peak current value. Hence, PBS with pH value of 4.0 was selected for the analysis that followed. Furthermore, as the electrolyte pH increased, the anodic peak potential (*E*_pa_) of BHA oxidation exhibited markedly negative movement, showing that protons were implicated in the electro-oxidation of BHA. The linear formula for the relationship between pH and the oxidation peak potential of BHA is: *E*_pa_(V) = −0.0572 pH + 0.6832 (R^2^ = 0.9949). The peak potential and pH are related by:

$$\frac{dE_{p}}{dpH}=\frac{2.303mRT}{nF}$$

Substituting the equivalency of the slope values of the *E*_p_ versus pH plot resulted in an *m*/*n* ratio of 1, showing that the process involved an equal number of protons and electrons [34].

CV experiments at various scan speeds were carried out to investigate the electrochemical redox reaction kinetics of BHA on FePor−COF−366/GCE surfaces. Figure 5B indicates how the peak current grew progressively as the scan rate increased between 25 and 350 mV s^−1^. The anodic and cathodic peak currents and the square root of the scan rates were linearly related, and followed the equations: *I*_pa_ (μA) = −0.3314 + 0.3415 ν^1/2^, (R^2^ = 0.9986); *I*_pc_ (μA) = −0.1525 − 0.0737 ν^1/2^, (R^2^ = 0.9834) (Inset a in Figure 5B). Diffusion was therefore primarily in charge of controlling the redox reaction process. The reduction peak potential (*E*_pc_) shifted in a more negative direction as the scan rate increased, while the oxidation peak potential (*E*_pa_) steadily moved in a more positive direction. The peak potentials and lnν can be represented by the linear equations: *E*_pa_ (V) = 0.4346 + 0.0163 lnν, (R^2^ = 0.9967); *E*_pc_ (V) = 0.0812 − 0.0216 lnν, (R^2^ = 0.9833) (Inset b in Figure 5B). The Laviron equation states:

(1) $$E_{\mathrm{pa}}=E^{\theta }+\left(\frac{\mathrm{RT}}{\alpha \mathrm{nF}}\right)\ln v$$

(2) $$E_{\mathrm{pc}}=E^{\theta }-\left[\frac{\mathrm{RT}}{\left(1-\alpha \right)\mathrm{nF}}\right]\ln v$$

**Figure 6:**
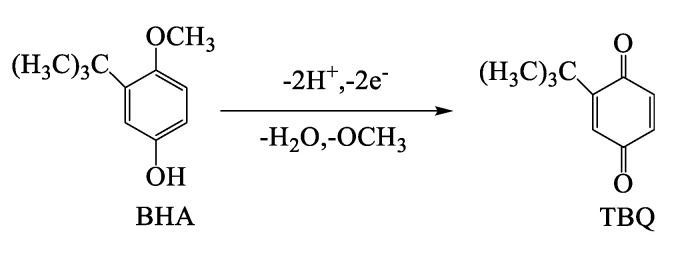
Electrochemical oxidation mechanism of BHA.

The linear slopes of *E*_pa_ and *E*_pc_ relative to lnν are RT/αnF and RT/((1 − α)nF), respectively. n can be calculated as 2.1. Because the oxidation of BHA is a two-electron transfer process, two electrons and two protons are included in the redox reaction [35]. The reaction mechanism is shown in Figure 6.

**Figure 7:**
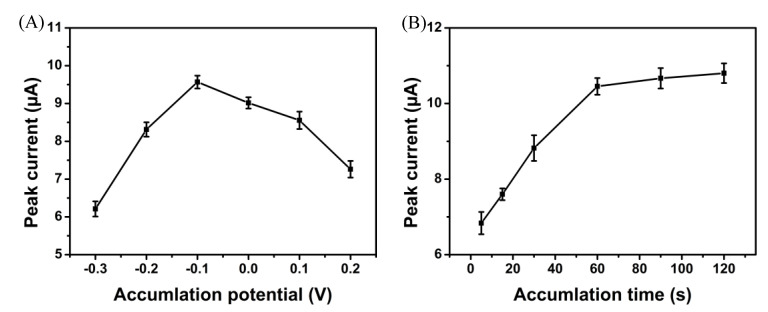
Influence of (**A**) accumulation potential and (**B**) time on the peak currents of BHA.

Furthermore, the accumulation step can be considered an effective method for improving the assay sensitivity. As shown in Figure 7A, the influence of accumulated potential on the peak current of BHA was tested from −0.3 to 0.2 V. The peak current reached its maximum value at −0.1 V, indicating that −0.1 V was favourable for the detection of BHA. As indicated in Figure 7B, considering the accumulation time, the peak current rose and reached its highest value at 60 s. As the amount of time increased, saturation of the BHA molecules occurred on the modified electrode. Therefore, 60 s was selected for subsequent studies.

### 3.4. Analytical Performance

**Figure 8:**
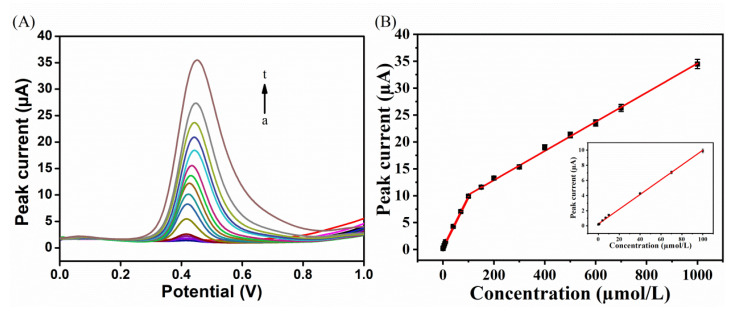
(**A**) DPV values for FePor−COF−366/GCE detection of BHA with different concentrations (from a to t: 0.04, 0.07, 0.1, 0.4, 0.7, 1.0, 4.0, 7.0, 10, 40, 70, 100, 150, 200, 300, 400, 500, 600, 700, 1000 μM) in 0.1 M pH 4.0 PBS. (**B**) Calibration plot of peak current versus BHA concentrations. Inset: trend of current intensity at low BHA concentrations.

DPV was applied under optimal conditions to study the relationship between peak current and BHA concentration (Figure 8A). According to the linear regressions illustrated in Figure 8B, the peak currents of BHA were proportional to its concentrations between 0.04–100 and 100–1000 μM, with the linear regressions: *I*_pa_ (µA) = 0.3415 C − 0.3314 (C: 0.04–100 μM, R^2^ = 0.998) and *I*_pa_ (µA) = 0.1525 C − 0.0737 (C: 100–1000 μM, R^2^ = 0.997). The two linear regions in Figure 8B can be explained as follows: when the concentration of BHA was low, BHA was deposited on the electrode surface; at this time, the electrochemical oxidation process was mainly controlled by the adsorption process, and BHA molecules on the electrode surface were rapidly transformed. With the increase of BHA concentration, more BHA molecules were deposited on the surface of the modified electrode, and the mass transfer resistance increased, leading to the decrease of sensitivity. The detection limit was calculated as 0.015 μM, using a signal-to-noise ratio of 3 (S/N).

According to the data in Table 1, comparing the performance of FePor−COF−366/GCE with other BHA sensors, FePor−COF−366/GCE had the lowest detection limit and the widest linear region. Several factors could account for its high analytical performance: (1) The metal porphyrins exist in a monomolecular state through the connection of metal ions, so that the metal ions are separated from each other and avoid aggregation, thereby increasing the catalytic active site and improving the catalytic activity; (2) the rigid and conjugated structure of Por-COFs at the molecular level endows them with inherent porous properties, enabling them to adsorb more BHA molecules; (3) Por-COFs have abundant π electron conjugated macrocycles, which can enhance affinity for BHA by π–π stacking and hydrogen bonding.

### 3.5. Interference, Stability, and BHA Detection

A number of potential coexisting substances were evaluated by DPV in 50 μM BHA, to examine the selectivity of the sensor. These experiments showed that many inorganic ions, including Zn^2+^, Mn^2+^, Mg^2+^, Cl^−^, and SO_4_^2−^, did not obstruct the measurement of BHA. Furthermore, the BHA measurement was unaffected by ascorbic acid, glucose, and L-cysteine concentrations that were 20-fold greater. Butylated hydroxytoluene (BHT) is another phenolic antioxidant which shares the same hydroxyl group as BHA and can be oxidized close to the potential of BHA. It was found that BHT had no impact on the detection of BHA, even at a 20-fold concentration. Together, these findings demonstrate the great selectivity of the electrochemical sensor for BHA detection. Storage stability was assessed by storing a batch of freshly manufactured electrodes in a refrigerator at 4 °C and taking measurements every three days. After a lengthy storage period of 21 days, it was discovered that approximately 91.2% of the initial value was maintained. The outcomes amply demonstrated the sensor’s ideal stability. Moreover, there was no evident decrease of current response observed after 35 cycles, and 95% of its original value was maintained. Three parallel measurements were taken for each sample, using the standard addition method, to assess the usefulness of the constructed electrochemical sensor. Table 2 showed that the recoveries ranged from 98.4 to 102.2%, and the RSD values ranged from 1.5% to 3.4%, indicating that the electrochemical sensor was capable of detecting the presence of BHA in actual samples.

## 4. Conclusions

In conclusion, the pre-designed Por-COFs were successfully used as metallization modification matrixes, and the metal atoms were regulated in the form of coordinated metal-N4 as special functional modulators. The resulting FePor-COFs demonstrated distinctly higher electrocatalytic response for BHA in terms of wide linear range, low detection limit, high sensitivity, superior selectivity, and good reproducibility. This study not only describes an attractive and efficient catalyst electrode for BHA determination, but also opens a new avenue for the design and development of two-dimensional porphyrin COFs as sensors for electrochemical detection of small molecules.

## Figures and Tables

**Table 1:** Comparison of this work with other electrochemical materials for BHA detection.

Electrode Materials	Line Range (μM)	Detection Limit (μM)	Ref.
Poly carminic acid/MWCNT ^1^	0.25–75	0.23	[36]
AuNPs ^2^/GCE	0.55–8.3	0.22	[37]
SPE–MWCNT ^3^	0.5–10	0.18	[38]
GCE/IrOxNPs ^4^	1–280	0.60	[39]
ZnO TPHS ^5^@GO ^6^ hybrid/GCE	0.3–60	0.04	[40]
Au-NP/Graphite	3.3–400	0.5	[41]
Surfactant/CPE ^7^	1.1–10.2	0.07	[42]
Core-shell MIP ^8^/GCE	0.6–300	1.62	[43]
FePor−COF−366/GCE	0.04–1000	0.015	This Work

^1^ MWCNT: multiwalled carbon nanotube, ^2^ AuNPs: gold nanoparticles, ^3^ SPE-MWCNT: multi-walled carbon nanotube modified screen-printed electrodes, ^4^ IrOxNPs: iridium oxide nanoparticles, ^5^ TPHS: hierarchical triple-shelled porous hollow spheres, ^6^ GO: graphene oxide, ^7^ CPE: carbon paste electrode, ^8^ MIP: molecularly imprinted polymer.

**Table 2:** Determination of BHA in real samples by FePor−COF−366/GCE.

Samples	Added (μM)	Obtained (μM)	RSD (%)	Recovery (%)
Peanut oil	0	Not detected	-	-
	5.0	4.98	2.8	99.6
	50.0	50.6	3.4	101.2
Rapeseed oil	0	Not detected	-	-
	5.0	4.92	1.5	98.4
	50.0	49.4	2.3	100.4
Corn oil	0	Not detected	-	-
	5.0	5.11	3.0	102.2
	50.0	49.4	2.1	98.8

## Data Availability

Not available.

## References

[1] Wang P., Han C.Y., Zhou F.Y., Lu J.S., Han X.G., Wang Z.W. (2016). Electrochemical determination of tert-butylhydroquinone and butylated hydroxyanisole at choline functionalized film supported graphene interface. Sens. Actuators B Chem..

[2] Wang L., Yang R., Wang H., Li J.J., Qu L.B., Harrington P.B. (2015). High-selective and sensitive voltammetric sensor for butylated hydroxyanisole based on AuNPs-PVP-graphene nanocomposites. Talanta.

[3] Yue X.Y., Song W.S., Zhu W.X., Wang J.L., Wang Y.R. (2015). In situ surface electrochemical co-reduction route towards controllable construction of AuNPs/ERGO electrochemical sensing platform for simultaneous determination of BHA and TBHQ. Electrochim. Acta.

[4] Freitas K.H., Fatibello-Filho O. (2010). Simultaneous determination of butylated hydroxyanisole (BHA) and butylated hydroxytoluene (BHT) in food samples using a carbon composite electrode modified with Cu_3_(PO_4_)_2_ immobilized in polyester resin. Talanta.

[5] Manoranjitham J.J., Narayanan S.S. (2021). Electrochemical sensor for determination of butylated hydroxyanisole (BHA) in food products using poly O-cresolphthalein complexone coated multiwalledcarbon nanotubes electrode. Food Chem..

[6] Delgado-Zamarreño M.M., González-Maza I., Sánchez-Pérez A., Carabias Martínez R. (2007). Analysis of synthetic phenolic antioxidants in edible oils by micellar electrokinetic capillary chromatography. Food Chem..

[7] Akkbik M., Assim Z.B., Ahmad F.B. (2011). Optimization and Validation of RP-HPLC-UV/Vis Method for Determination Phenolic Compounds in Several Personal Care Products. Int. J. Anal. Chem..

[8] Chen M., Hu X.J., Tai Z.G., Qin H., Tang H.N., Liu M.S., Yang Y.L. (2012). Determination of Four Synthetic Phenolic Antioxidants in Edible Oils by High-Performance Liquid Chromatography with Cloud Point Extraction Using Tergitol TMN-6. Food Anal Methods.

[9] Davoli E., Bastone A., Bianchi G., Salmona M., Diomede L. (2017). A simple headspace gas chromatography/mass spectrometry method for the quantitative determination of the release of the antioxidants butylated hydroxyanisole and butylated hydroxytoluene from chewing gum. Rapid Commun. Mass Spectrom..

[10] Capitan-Vallvey L.F., Valencia M.C., Nicolas E.A. (2001). Monoparameter sensors for the determination of the antioxidants butylated hydroxyanisole and n-propyl gallate in foods and cosmetics by flow injection spectrophotometry. Analyst.

[11] Shu Y., Li B., Xu Q., Gu P., Xiao X., Liu F.P., Yu L.Y., Pang H., Hu X.Y. (2017). Cube-like CoSn(OH)_6_ nanostructure for sensitive electrochemical detection of H_2_O_2_ in human serum sample. Sens. Actuators B Chem..

[12] Wang J., Li N., Xu Y.X., Pang H. (2020). Two-Dimensional MOF and COF Nanosheets: Synthesis and Applications in Electrochemistry. Chem. Eur. J..

[13] Chen Y.L., Xie Y., Sun X., Wang Y., Wang Y. (2021). Tunable construction of crystalline and shape-tailored Co_3_O_4_@TAPB-DMTP-COF composites for the enhancement of tert-butylhydroquinone electrocatalysis. Sens. Actuators B Chem..

[14] Wang J., Xu Q., Xia W.W., Shu Y., Jin D.Q., Zang Y., Hu X.Y. (2018). High sensitive visible light photoelectrochemical sensor based on in-situ prepared flexible Sn_3_O_4_ nanosheets and molecularly imprinted polymers. Sens. Actuators B Chem..

[15] Zhu R.M., Ding J.W., Jin L., Pang H. (2019). Interpenetrated structures appeared in supramolecular cages, MOFs, COFs. Coord. Chem. Rev..

[16] Huang Z.L., Xu Q., Hu X.Y. (2020). Covalent organic frameworks functionalized carbon fiber paper for the capture and detection of hydroxyl radical in the atmosphere. Chin. Chem. Lett.

[17] Zhang T., Chen Y.L., Huang W., Wang Y., Hu X.Y. (2018). A novel AuNPs-doped COFs composite as electrochemical probe for chlorogenic acid detection with enhanced sensitivity and stability. Sens. Actuators B Chem..

[18] Xie Y., Zhang T., Chen Y.L., Wang Y., Wang L. (2020). Fabrication of core-shell magnetic covalent organic frameworks composites and their application for highly sensitive detection of luteolin. Talanta.

[19] Sun Y., Chen C.Y., Liu J.B., Liu L.Z., Tuo W., Zhu H.T.Z., Lu S., Li X.P., Stang P.J. (2020). Self-Assembly of Porphyrin-Based Metallacages into Octahedra. J. Am. Chem. Soc..

[20] Cai W.R., Zeng H.B., Xue H.G., Marks R.S., Cosnier S., Zhang X.J., Shan D. (2020). Enhanced Electrochemiluminescence of Porphyrin-Based Metal-Organic Frameworks Controlled via Coordination Modulation. Anal. Chem..

[21] Liu M.J., Cao S.M., Feng B.Q., Dong B.X., Ding Y.X., Zheng Q.H., Teng Y.L., Li Z.W., Liu W.L., Feng L.G. (2020). Revealing the structure-activity relationship of two Cu-porphyrin-based metal-organic frameworks for the electrochemical CO_2_-to-HCOOH transformation. Dalton Trans..

[22] Wu L.T., Han C., Wang Z.J., Wu X., Su F., Li M.Y., Zhang Q.Y., Jing X.B. (2020). Porphyrin-Based Organoplatinum(II) Metallacycles With Enhanced Photooxidization Reactivity. Front Chem..

[23] Chen R.F., Wang Y., Ma Y., Mal A., Gao X.Y., Gao L., Qiao L.J., Li X.B., Wu L.Z., Wang C. (2021). Rational design of isostructural 2D porphyrin-based covalent organic frameworks for tunable photocatalytic hydrogen evolution. Nat. Commun..

[24] Wan S., Gándara F., Asano A., Furukawa H., Saeki A., Dey S.K., Liao L., Ambrogio M.W., Botros Y.Y., Duan X.F. (2011). Covalent Organic Frameworks with High Charge Carrier Mobility. Chem. Mater..

[25] Wang D.W., Zhang Z., Lin L., Liu F., Wang Y.B., Guo Z.P., Li Y.H., Tian H.Y., Chen X.S. (2019). Porphyrin-based covalent organic framework nanoparticles for photoacoustic imaging-guided photodynamic and photothermal combination cancer therapy. Biomaterials.

[26] Meng F.L., Qian H.L., Yan X.P. (2021). Conjugation-regulating synthesis of high photosensitizing activity porphyrin-based covalent organic frameworks for photodynamic inactivation of bacteria. Talanta.

[27] Xu Q.T., Xue H.G., Guo S.P. (2018). FeS2 walnut-like microspheres wrapped with rGO as anode material for high-capacity and long-cycle lithium-ion batteries. Electrochim. Acta.

[28] Dai L.X., Li W.L., Zhou K.H., Tang D.M., Han Y., Wu X.Y., Wu H.Y., Diao G.W., Chen M. (2019). Interfacial anchoring effect for enhanced lithium storage performance of sesame balls-like Fe_3_O_4_/C hollow nanospheres. J. Electroanal. Chem..

[29] Shu Y., Xu J., Chen J.Y., Xu Q., Xiao X., Jin D.Q., Pang H., Hu X.Y. (2017). Ultrasensitive electrochemical detection of H_2_O_2_ in living cells based on ultrathin MnO_2_ nanosheets. Sens. Actuators B Chem..

[30] Wei C.Z., Cheng C., Ma L., Liu M.N., Kong D.C., Du W.M., Pang H. (2016). Mesoporous hybrid NiO_x_-MnO_x_ nanoprisms for flexible solid-state asymmetric supercapacitors. Dalton Trans..

[31] Gao Y.J., Yang F.Y., Yu Q.H., Fan R., Yang M., Rao S.Q., Lan Q.C., Yang Z.J., Yang Z.Q. (2019). Three-dimensional porous Cu@Cu_2_O aerogels for direct voltammetric sensing of glucose. Mikrochim Acta.

[32] He H., Dong J., Li K., Zhou M., Xia W.W., Shen X.S., Han J.R., Zeng X.H., Cai W.P. (2015). Quantum dot-assembled mesoporous CuO nanospheres based on laser ablation in water. RSC Adv..

[33] Jiang J.J., Ding D., Wang J., Lin X.Y., Diao G.W. (2021). Three-dimensional nitrogen-doped graphene-based metal-free electrochemical sensors for simultaneous determination of ascorbic acid, dopamine, uric acid, and acetaminophen. Analyst.

[34] Mao A.R., Li H.B., Yu L.Y., Hu X.Y. (2017). Electrochemical sensor based on multi-walled carbon nanotubes and chitosan-nickel complex for sensitive determination of metronidazole. J. Electroanal. Chem..

[35] Yang C., Liu M.M., Bai F.Q., Guo Z.Z., Liu H., Zhong G.X., Peng H.P., Chen W., Lin X.H., Lei Y. (2019). An electrochemical biosensor for sensitive detection of nicotine-induced dopamine secreted by PC12 cells. J. Electroanal. Chem..

[36] Ziyatdinova G., Guss E., Budnikov H. (2020). Amperometric sensor based on MWNT and electropolymerized carminic acid for the simultaneous quantification of TBHQ and BHA. J. Electroanal. Chem..

[37] Lin X.Y., Ni Y.N., Kokot S. (2013). Glassy carbon electrodes modified with gold nanoparticles for the simultaneous determination of three food antioxidants. Anal. Chim. Acta.

[38] Caramit R.P., de Freitas Andrade A.G., Gomes de Souza J.B., de Araujo T.A., Viana L.H., Trindade M.A.G., Ferreira V.S. (2013). A new voltammetric method for the simultaneous determination of the antioxidants TBHQ and BHA in biodiesel using multi-walled carbon nanotube screen-printed electrodes. Fuel.

[39] Roushani M., Sarabaegi M. (2014). Electrochemical detection of butylated hydroxyanisole based on glassy carbon electrode modified by iridium oxide nanoparticles. J. Electroanal. Chem..

[40] Gan T., Zhao A.X., Wang S.H., Lv Z., Sun J.Y. (2016). Hierarchical triple-shelled porous hollow zinc oxide spheres wrapped in graphene oxide as efficient sensor material for simultaneous electrochemical determination of synthetic antioxidants in vegetable oil. Sens. Actuators B Chem..

[41] Ng K.L., Tan G.H., Khor S.M. (2017). Graphite nanocomposites sensor for multiplex detection of antioxidants in food. Food Chem..

[42] De Araujo T.A., Barbosa A.M., Viana L.H., Ferreira V.S. (2010). Voltammetric determination of tert-butylhydroquinone in biodiesel using a carbon paste electrode in the presence of surfactant. Colloids Surf. B.

[43] Zhao P.N., Hao J.C. (2013). Tert-butylhydroquinone recognition of molecular imprinting electrochemical sensor based on core-shell nanoparticles. Food Chem..

